# Precancer antiviral treatment reduces microvascular invasion of early-stage Hepatitis B-related hepatocellular carcinoma

**DOI:** 10.1038/s41598-019-39440-7

**Published:** 2019-02-18

**Authors:** Kai Liu, Jicheng Duan, Hu Liu, Xinwei Yang, Jiahe Yang, Mengchao Wu, Yanxin Chang

**Affiliations:** Department of Surgery, Eastern Hepatobiliary Surgery Hospital, Second Military Medical University, Shanghai, 200438 PR China

**Keywords:** Cancer therapy, Risk factors

## Abstract

The impact of antiviral therapy before tumorigenesis on microvascular invasion (MVI) of Chronic hepatitis B (CHB)-related hepatocellular carcinoma (HCC) is still unknown. In this retrospective cohort study 3,276 HCC patients with early-stage who underwent curative resection were included. We investigated the effect of precancer antiviral therapy on the pathology, especially MVI, of CHB-related HCC. MVI occurrence rates of CHB-related HCC stratified by histopathologic inflammation grades of G1, G2, and G3 were 30.4%, 34.7%, and 38.6%, respectively, compared to 19.8% for CHB-negative HCC. Patients who received standard antiviral treatment showed much lower rates of MVI, higher tumor capsule integrity, less frequent satellite micronodules and lower AFP level compared to the no antiviral group. Moreover, precancer antiviral therapy prolonged the disease-free survival (DFS), which are also proved to be independent indicators of DFS. In addition, we show that antivirals may suppress early progression of HCC primarily by inhibition of HBV viral load, and influencing the expression levels of CK18, GPC3, OPN and pERK. Hence, we demonstrate that precancer antivirals significantly reduce the MVI rate of CHB-related HCC, reduce malignancy of early-stage HCC, and improve HCC prognosis. Thus, this study confirms the importance of antiviral therapy for CHB patients.

## Introduction

Chronic hepatitis B (CHB) is the predominant risk factor for carcinogenesis and progression of hepatocellular carcinoma (HCC), accounting for approximately 50% of all HCC cases. The etiologic mechanisms of CHB-related HCC are thought to involve several factors inducing liver fibrogenesis, genetic mutations, and the expression and action of active viral-encoded proteins. Fibrosis and cirrhosis, resulting from CHB-associated persistent liver inflammation, trigger a complex cascade of oxidative stress, hypoxia, necrosis, regeneration and angiogenesis, which may alter host gene expression over a period of years^1^.

Liver resection, transplantation and radiofrequency ablation therapy are considered to be curative treatments for HCC. However, the long-term outcomes of HCC patients remain unsatisfactory due to high rates of intra- and extra-hepatic recurrence^2^. Many studies have demonstrated that effective antiviral treatment using nucleotide/nucleoside analogs (NAs) not only prevent the incidence of HCC in CHB patients, but also reduce or delay HCC recurrence and finally improve the prognosis of HCC^3–6^. Therefore, the significant benefits of antiviral therapy in patients with HBV-related HCC should be emphasized.

Microvascular invasion (MVI) has been extensively demonstrated as an independent risk factor for adverse outcomes such as early recurrence following curative liver resection or transplantation in HCC patients. It was reported that the recurrence-free survival (RFS) rates at 2 years post-operation, in patients without MVI and with MVI, were 75.9% and 32.7%, respectively^7^. Thus, MVI is a significant prognostic factor for HCC. Although, MVI is difficult to detect before surgical treatment, Shen *et al*.^8^ has established a nomogram for preoperative estimation of MVI in CHB-related HCC, which indicates that high DNA load (>10^4^ IU/mL) is independently associated with MVI. Since MVI is a common event in advanced HCC^9^, the evaluation of MVI occurrence is more valuable at early stages. However, the relationship and mechanisms between antiviral treatment before tumorigenesis (i.e. precancer antiviral therapy) and MVI occurrence in CHB-related HCC, especially at the early stage, are still under studied. Consequently, the purpose of this retrospective cohort study was to explore the influence of precancer antiviral treatment on HCC and to investigate whether it reduces the occurrence of MVI in early-stage HCC based on the BCLC (Barcelona Clinic Liver Cancer) and Milan staging system^10,11^.

## Results

### MVI and CHB inflammation

**Figure 1:**
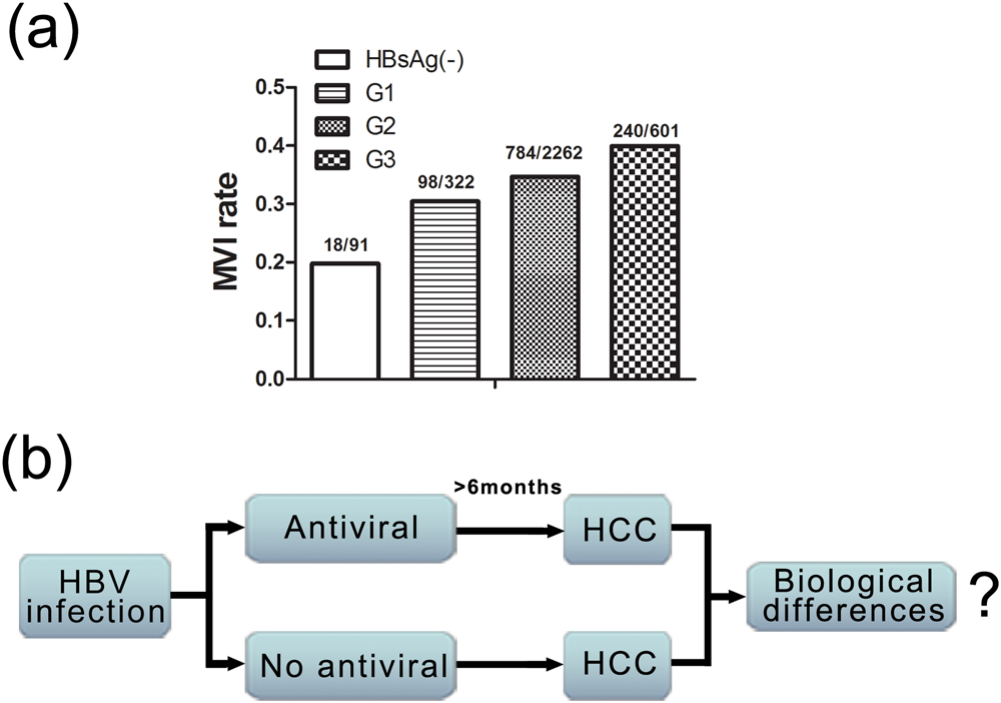
MVI rate is closely related with histopathologic inflammation grades. (**a**) The relationship between MVI and GS grade of HCC. (**b**) The schematic depiction of the design of this study.

To clarify the role of HBV-related inflammation in HCC, we investigated the prevalence of MVI which is associated with different histopathologic inflammation grades of HCC. We found that, of the 3,276 total HCC patients (Table 1, Supplementary Information) of early-stage according to BCLC and Milan criteria, defined as a single HCC ≤ 5 cm in the maximum diameter, MVI was detected in 30.4% (98/322), 34.7% (784/2262), and 39.9% (240/601) of tumors with histopathologic grades of G1, G2, and G3, respectively. In contrast, the MVI rate in HBsAg negative HCC patients was only 19.8% (18/91) (Fig. 1a). The differences between the G1, G2, and G3 groups and the HBsAg(−) group were all significant (G1 vs HBsAg(−), *P* < 0.05; G2 vs HBsAg(−), *P* < 0.01; G3 vs HBsAg(−), *P* < 0.01). Importantly, higher histopathologic inflammation grades were associated with higher MVI rates, with statistical significance (G3 vs G1, *P* < 0.01; G3 vs G2, *P* < 0.05; G2 vs G1, *P* = 0.13). This provides strong evidence that HBV-related inflammation is implicated in MVI occurrence. Considering the importance of MVI in HCC progression, especially on recurrence and metastasis, inhibition of such inflammation may improve the prognosis of HCC patients.

**Table 1:** Summary of Clinicopathologic Variables.

Characteristic	Number of Patients
Patients	3276 (include 91 HBsAg(−))
Sex	
male	2832
female	444
Age (years)	17–87, median = 53
Tumor size (cm)	1.2–5.0, median = 3.5
GS staging	
HBsAg(−)	91
G1	322
G2	2262
G3	601

The presence of MVI significantly worsens the surgical outcomes of early-stage HCC. As yet, there has been no study that has directly investigated the relationship between precancer antiviral therapy and MVI. To address this, we designed this retrospective cohort study according to the schema shown in Fig. 1b. CHB-related HCC patients were divided into two groups: one which received antiviral therapy for at least 6 months, meanwhile at the beginning it was confirmed that there were no tumors detected by an imaging examination before the initial NAs were administered, and another which received no treatment. After follow-up, CHB patients were diagnosed for HCC and received surgical resection, after which the clinical data were collected and analyzed. Based on such criteria, out of 3,276 total CHB patients, 349 consecutive patients (139 in antiviral group, 119 in no antiviral group, 91 in HBsAg negative group for control) were finally enrolled in this study (More detailed information can be found in Materials and methods).

### Effect of precancer antiviral therapy on MVI occurrence

**Figure 2:**
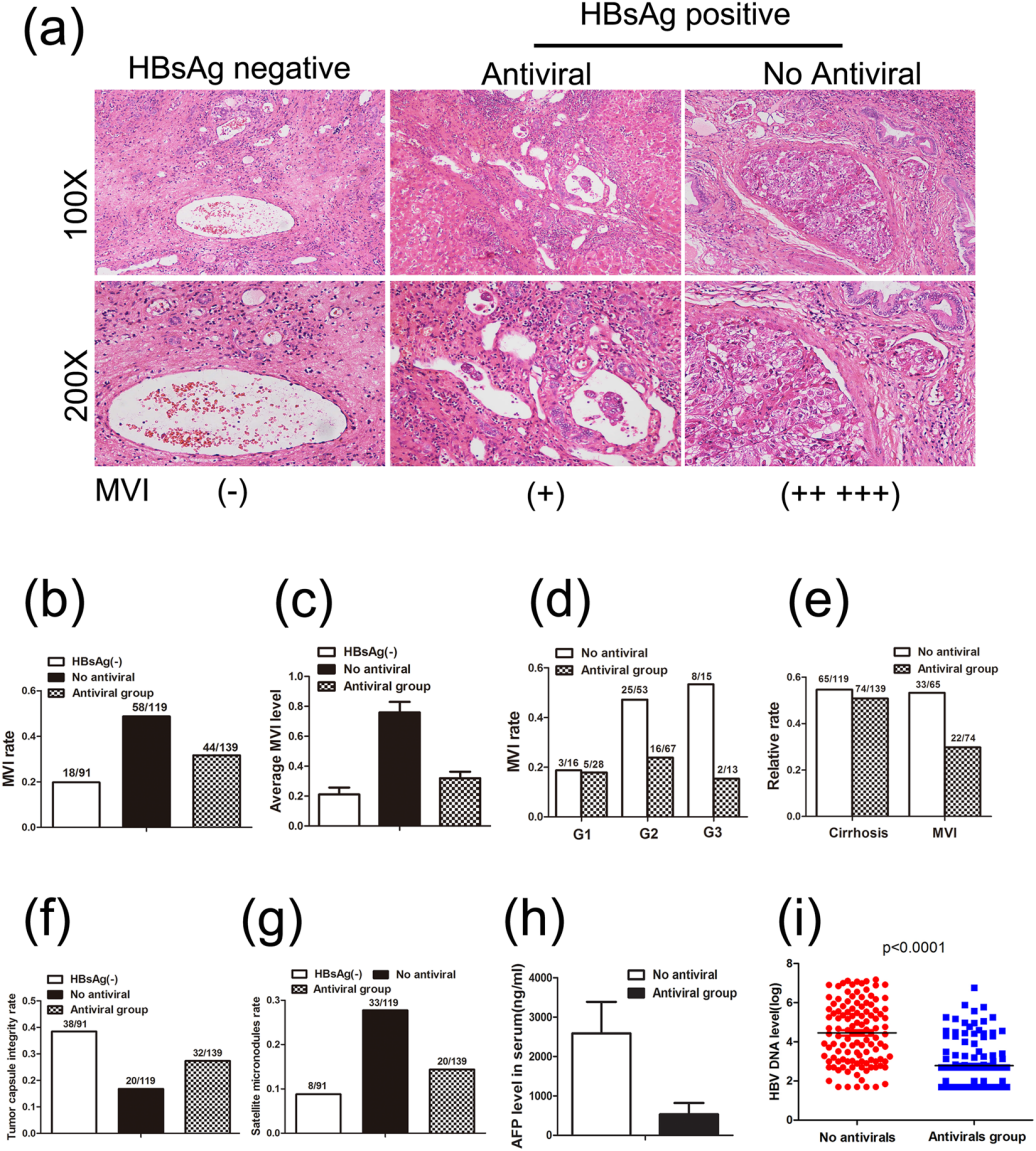
Antiviral therapy improves pathologic characteristics, especially MVI occurrence. (**a)** Examples of MVI in antiviral and no antiviral groups, compared to the HBsAg(−) group. (**b)** MVI occurrence rate in antiviral and no antiviral groups compared to the HBsAg(−) group. **(c)** Average level of MVI in antiviral and no antiviral groups compared to the HBsAg(−) group. **(d)** Difference in MVI occurrence rates between antiviral and no antiviral groups stratified by histopathologic inflammation grade (G1/G2/G3). **(e)** Difference in MVI occurrence rates between antiviral and no antiviral groups among cirrhosis patients. (**f**) Tumor capsule integrity tare in antiviral and no antiviral groups compared to the HBsAg(−) group. (**g**) Satellite micronodules rate in antiviral and no antiviral groups compared to the HBsAg(−) group. (**h**) Difference in serum AFP levels between antiviral and no antiviral groups. (**i**) Difference in HBV DNA loads between antiviral and no antiviral groups.

To explore the effect of precancer antiviral therapy on MVI occurrence in HCC, we focused on the pathological data of 349 patients under curative resection. As shown in Fig. 2a,b, the MVI rate of the HBsAg negative group was 19.8%, whereas the MVI rate in the CHB group without antiviral therapy was much higher (48.7% vs 19.8%, *P* < 0.0001), indicating that MVI is closely correlated with HBV infection. In addition, as shown in Fig. 2c, the average level of MVI was much lower in the antiviral group compared to the no antiviral group (0.32 vs 0.76, *P* < 0.0001), but slightly higher than in patients without HBV infection (0.21). Additionally, the differences in average MVI levels between the antiviral and no antiviral groups were much more pronounced when stratified by histopathologic grade, but only at G2 and G3 grades (Fig. 2d). Along with the CHB-associated inflammation, cirrhosis is the common complication of CHB. Although the percentage of cirrhosis patients in antiviral group (50.8%, 74/139) was similar to that of no antiviral group (54.6%, 65/119), antiviral therapy could still obviously decreased the MVI rate of cirrhosis patients (29.7% vs 50.8%, *P* < 0.05, Fig. 2e). It has been reported that several factors influence the occurrence of MVI, such as tumor number, HBV DNA level, and serum α-fetoprotein (AFP)^8^. We then discovered that the antiviral group exhibited a much lower MVI rate (31.7% vs 48.7%, *P* < 0.01), a higher tumor capsule integrity (27.3% vs 16.8%, *P* < 0.05, Fig. 2f), fewer satellite micronodules (14.4% vs 27.7%, *P* < 0.01, Fig. 2g), and lower level of serum AFP level (2587 ± 800 vs 533 ± 289 ng/ml, *P* < 0.01, Fig. 2h), compared to the no antiviral group. These results fully illustrate the importance of precancer antiviral therapy in decreasing the occurrence of MVI, especially in the presence of a large amounts of HBV-induced inflammation. This suggests that even in CHB-related HCC patients with G3 grade or cirrhosis, antiviral therapy could still decrease the malignancy of tumors.

We then further investigated the HBV DNA load of the two groups, and discovered that patients receiving antiviral therapy for more than 6 months had a much lower log mean HBV DNA load (2.78 vs 4.45, *P* < 0.0001, Fig. 2i). It has been reported that a log HBV DNA load greater than 4 is an independent preoperative indicating factor associated with MVI occurrence^9^. Antivirals inhibit hepatitis and protect liver function mainly by reducing HBV viral load. Thus, a reduced amount of HBV DNA is expected to correlate with milder HBV-related inflammation. This may be the major reason for reducing preoperative MVI occurrence in HCC patients.

### Relationship between precancer antiviral therapy and disease-free survival of patients

**Figure 3:**
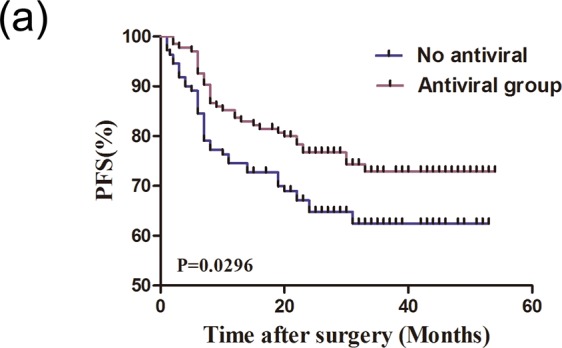
Antiviral therapy improves the prognosis of patients. (**a**) Kaplan-Meier analysis of disease free survival (DFS) rates in HCC patients treated with or without antivirals.

A retrospective disease-free survival (DFS) analysis was carried out to further determine the clinical significance of antiviral therapy before tumorigenesis in the development of HCC. As shown in Fig. 3a, 139 patients who received precancer antiviral therapy exhibited much longer DFS than the control group (mean DFS time were 33 and 11 months, respectively, difference = 22 months, *P* < 0.05). Furthermore, univariable and multivariable Cox regression analysis (Table 2) confirmed that precancer antiviral therapy (Hazard Ratio = 0.635, 95% CI: 0.460–0.876, *P* < 0.01), including MVI (Hazard Ratio = 1.703, 95% CI: 1.183–2.452, *P* < 0.01) and tumor satellite micronodules (Hazard Ratio = 0.621, 95% CI: 0.425–0.910, *P* < 0.05), to be independent indicators of DFS. This further reinforces the key role that antiviral therapy plays in delaying or preventing tumor invasion and metastasis. More importantly, this result indicates that HCC in CHB patients receiving regular antiviral therapy will likely have better prognosis after surgery even if they do not receive adjunctive therapies, such as TACE. The results also suggest that antiviral therapy could be an independent indicator of DFS.

**Table 2:** The univariable and multivariable Cox regression analysis for the disease-free survival.

Clinical variable	Univariate			Multivariate		
	HR	CI	*P*	HR	CI	*P*
**Sex** Male or Female	0.922	0.602–1.412	0.707	0.903	0.575–1.418	0.657
**Age (years)** ≤51 or >51	1.074	0.796–1.449	0.642	1.250	0.909–1.717	0.169
**Antiviral therapy** Yes or No	0.736	0.5440–0.995	**0.046**	0.635	0.460–0.876	**0.006**
**MVI** Yes or No	1.541	1.091–2.176	**0.014**	1.703	1.183–2.452	**0.004**
**Tumor size (cm)** ≤3 or >3	1.138	0.843–1.536	0.397	1.156	0.583–1.216	0.357
**Tumor capsule integrity** Yes or No	0.829	0.580–1.184	0.303	0.842	0.576–1.200	0.359
**Tumor satellite micronodules** Yes or No	0.735	0.521–1.037	0.080	0.621	0.425–0.910	**0**.**014**

CI = confidence interval, P values are from univariate or multivariate Cox regression.

### Potential molecular mechanisms

The mechanisms of how antiviral therapy leads to a decrease in MVI occurrence are still unclear. To explore the underlying molecular mechanisms, we used IHC to thoroughly investigate the expression of related proteins that have been implicated in HCC, such as Hep-1 (Hepatocyte Paraffin 1), CK18 (cytokeratin-18), CK19 (Cytokeratin 19), CD34 (Cluster Of Differentiation 34), GPC-3 (Glypican-3), SUOX (sulfite oxidase), pERK (phosphorylation extracellular regulated protein kinases), OPN (osteopontin), Muc1 (mucin1), HSP70 (Heat Shock Protein 70), TRIM35 (tripartite motif containing 35), and PKM2 (Pyruvate kinase M2 isozyme)^12–22^.

**Figure 4:**
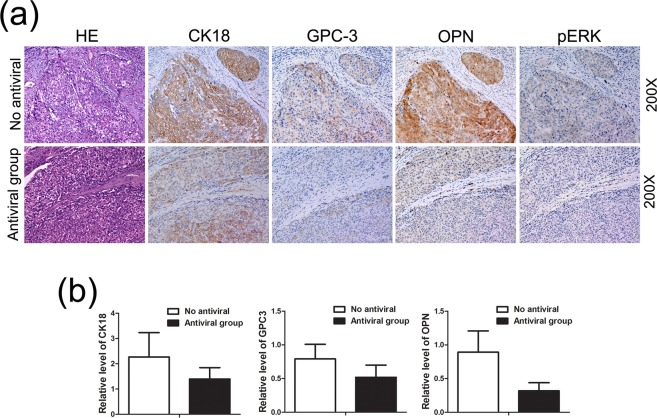
Potential mechanisms behind MVI reduction due to antiviral treatment. (**a**,**b**) Expression of CK-18, GPC-3, OPN, and pERK determined by immunohistochemistry assays.

After the IHC screening, as Fig. 4a shows, only CK18, GPC3, and OPN were expressed at a significantly higher level in the no antiviral HCC group compared to the antiviral group. It has been reported that the up-regulation of CK18, GPC3, and OPN are associated with increased tumor aggressiveness and poor prognosis^19,23–25^. Then, the IHC scoring was performed by adding the values of the intensity and relative abundance (Materials and Methods), and the average score of such proteins in each group was calculated. As shown in Fig. 4b, the differences are statistically significant, indicating that precancer antiviral therapy decreases MVI occurrence and tumor satellite micronodules, and improve the prognosis of HCC maybe by manipulating the expression of CK18, GPC3 or OPN. Thus, in HCC patients that do not receive antiviral therapy, the higher expression of these proteins may result in tumor recurrence and poorer prognosis. More importantly, pERK was found to be activated in the no antiviral HCC patients but not in the antiviral group, indicating that activation of the MAPK/ERK signaling pathway may be responsible for MVI occurrence, and also that suppression of HCC progression may be accomplished by manipulating this pathway. Meanwhile, more work will be needed for further understanding of potential mechanisms.

## Discussion

Cancer-related inflammation promotes neoplastic transformation and it has also been increasingly recognized as a major factor in tumor progression and metastasis. The serial progression of hepato-carcinogenesis occurs according to a sequence of inflammation, fibrosis, and then tumorigenesis, which is known as the hepatic inflammation-fibrosis-cancer axis (IFC axis). There is no doubt that HBV plays a key role in carcinogenesis and progression of HCC, therefore HBV antivirals are commonly used according to clinical guidelines for its prevention and treatment. It is possible that HBV also contributes to the malignancy of HCC.

HCC remains a major concern for CHB patients. The main goal of therapy is to improve survival and quality of life by preventing disease progression. Increasing numbers of studies have proved that antiviral treatment reduces HCC incidence in patients with CHB infection and improves RFS and overall survival (OS) rates of patients after curative treatment^26^. Concurrently, interferon therapy has failed to demonstrate a significant benefit as measured by RFS or OS rates^27^. Recently, the European Association for the Study of the Liver (EASL) has issued updated clinical practice guidelines for the management of HBV infection^28^. According to the most recent guidelines, patients who receive antiviral treatment would benefit from it. It is becoming increasingly clear that antiviral treatment is necessary due to the critical association between HBV DNA load and HCC carcinogenesis and development. Our study suggests that the more precise the therapeutic indications are, the greater the benefit will be to HBV patients, especially early-stage HCC patients. There are still many unresolved issues regarding guidelines for the initiation of antiviral therapy in patients with HBeAg-positive CHB, and for discontinuation in HBeAg-negative patients treated with NAs, which should be addressed in future studies.

It is well known that many factors influence the prognosis of HCC after curative resection, including tumor size, tumor number, HBV load, serum AFP level, platelet count, and MVI level. The presence of MVI is well known to correlate with a high frequency of short-term recurrence of HCC after liver resection and has also been demonstrated to be a strong and independent risk factor for poor outcome^7^. Our study, with a large sample size, focused on MVI of early-stage HCC and clarified that preoperative CHB-related inflammation and HBV load were the primary factors influencing the MVI level. A critical conclusion from this study is that inhibition of inflammation by NAs clearly decreased the MVI occurrence rate and significantly improved the prognosis of HCC after surgery, which adds further support to the classical inflammation-cancer theory. Furthermore, this data has revealed a promising target for intervention MAPK/ERK pathway and paves the way for future treatment of HCC.

There are some limitations to this study that should be pointed out. First, while our study is not a prospective study, the large sample size provides significant value. Second, although no literature exists that could be referred to for standard inclusion/exclusion criteria, we required that the time of antiviral treatment should be least 6 months, and ensured that tumors were absent upon initiation of antiviral treatment by a medical imaging examination. Third, due to the inherent limit on the follow-up time, complete information regarding OS was not available as this data was still being collected. Fourth, we focused on common molecular players instead of conducting high throughput screening using gene chips or sequencing to investigate the potential mechanisms behind the ability of antiviral therapy to decrease MVI occurrence. Thus, moving forward further studies are needed to address these limitations. Nevertheless, this study provides novel and meaningful data that contributes to a body of knowledge that will improve the management of HBV infections in the future.

In conclusion, the retrospective cohort study reveals that antiviral therapy significantly reduces the MVI occurrence rate of early-stage CHB-related HCC, and improves the prognosis of HCC. Moreover, the activation of the MAPK/ERK signaling pathway may be responsible for MVI occurrence, which needs more work for further mechanisms. These results are important and provide strong support for the necessity of antiviral therapy for CHB patients. Additionally, it provides new research directions for the improvement of the prognosis of CHB-related HCC.

## Materials and Methods

### Patients and Design

A total of 3,276 early-stage HCC patients, diagnosed according to the BCLC and Milan criteria, underwent curative liver resection at Eastern Hepatobiliary Surgery Hospital from 2013 to 2016, were enrolled in this study (Table 1). Of the 3,276 total patients, 3,185 had CHB-related HCC and 91 were HBsAg negative HCC. As the schema shows (Fig. 1c), the following criteria were used to select from the remaining 3,185 CHB-related HCC patients for further retrospective cohort study: (1) NAs (Lamivudine, Adefovir dipivoxil, Telbivudine or Entecavir) had been taken continuously for at least 6 months according to the hepatitis B guidelines; (2) There were no tumors detected by an imaging examination before the initial NAs were administered; (3) Patients received R0 resection; (4) Tumor maximum diameter was no more than 5 cm; (5) No macrovascular invasion was present; (6) Liver function assessed by Child-Pugh scoring was class A or B. After selection criteria were considered, 139 patients were enrolled into the “antiviral” group, and 119 were enrolled into the “no antiviral” group. Thus, including the 91 HBsAg(−) patients along with these two groups, 349 consecutive patients were selected for further study.

This study, including any relevant details, was approved by the local Institutional Review Board of the Second Military Medical University, and informed consent was obtained from all patients according to the regulations of the board. We confirm that all experiments were performed in accordance with relevant guidelines and regulations.

### Immunohistochemistry

All tissues obtained from surgical resections were fixed with 4% paraformaldehyde for at least 24 h and embedded in paraffin. Then 3μm parafin sections were put in 60 °C oven for overnight, then deparaffinized with xylene three times for 10 min, rehydrated with 100%, 95%, 80%, 70%, 50% ethanol (5 min each) successively and exposed to 3% H_2_O_2_ for 20 min at RT (room temperature) to block endogenous peroxidase activity. Then the sections were placed in a 0.01 M citrate buffer and heated at 120 °C for 2 min. 1% bovine serum albumin was used to block the nonspecific binding by preincubation with in 0.01 M PBS at RT for 30 min. The sections were incubated overnight at 4 °C with primary antibodies against CK18 (Abcam), GPC-3 (Abcam), OPN (Invitrogen), and pERK (Santa cruz), respectively. Next, the slides were taken out at RT for rewarming, then washed with 0.01 M PBS for three times (5 min each). A goat anti-rabbit IgG biotinylated antibody (Dako) was used as a secondary antibody for 30 min at RT. The color reaction was conducted in 2% diaminobenzidine in 50 mM Tris buffer for 30 sec. At last, the sections were counterstained with Meyer’s hematoxylin, dehydrated, and mounted with resin. All sections were examined independently by two pathological experts. The IHC scoring was performed under high power fields of 10 random tumor areas. The percentage of protein positive cells was graded from 0 to 4 (0 = less than 5% of positive cells; 1 = 5–25%; 2 = 26–50%; 3 = 51–75%; 4 = 76–100%). The intensity was graded from 0 to 3 and scored as follows: 0, equivalent to background staining of HCC; 1, weak staining; 2, moderate staining; and 3, intense staining. The composite score was calculated by adding the values of the intensity and relative abundance for further statistics^29^.

### Statistical Analysis

Numeration variables were expressed as the MVI grades. All data are presented as means or means ± standard deviations (SD). Statistical significance was calculated using Chi-square tests or unpaired Student’s t-tests. All tests were evaluated as two-tailed. Kaplan-Meier, log-rank and Cox regression analysis were used to assess the patient disease-free survival between subgroups.Statistical analysis was performed using SPSS software version 24.0 (SPSS Inc., Chicago, IL, USA). P values less than 0.05 were considered as statistically significant.

## Supplementary information

Dataset 1

## Data Availability

All data are fully available without restriction.
