# Publisher Correction: Precancer antiviral treatment reduces microvascular invasion of early-stage Hepatitis B-related hepatocellular carcinoma

**DOI:** 10.1038/s41598-019-43791-6

**Published:** 2019-05-17

**Authors:** Kai Liu, Jicheng Duan, Hu Liu, Xinwei Yang, Jiahe Yang, Mengchao Wu, Yanxin Chang

**Affiliations:** Department of Surgery, eastern Hepatobiliary Surgery Hospital, Second Military Medical University, Shanghai, 200438 PR China

Correction to: *Scientific Reports* 10.1038/s41598-019-39440-7, published online 18 February 2019

The original version of this Article contained an error in the Author information.

“Kai Liu, Xinwei Yang and Jiahe Yang contributed equally.”

now reads:

“Kai Liu, Jicheng Duan and Hu Liu contributed equally.”

This has now been corrected in the PDF and HTML versions of the paper.

